# The cage-free egg sector: perspectives of Indian poultry producers

**DOI:** 10.3389/fvets.2024.1442580

**Published:** 2024-08-07

**Authors:** Jaydip Rokade, Abhijeet Champati, Nagesh Sonale, Prasad Wadajkar, Monika Madheshwaran, Darshana Bhaisare, Ashok Kumar Tiwari

**Affiliations:** ^1^Division of Poultry Housing and Management, ICAR-Central Avian Research Institute, Izatnagar, Uttar Pradesh, India; ^2^Division of School of Animal Science, ICAR-Indian Agricultural Research Institute, Gauria Karma, Jharkhand, India; ^3^Department of Poultry Science, Nagpur Veterinary College, Nagpur, Maharashtra, India; ^4^ICAR-Central Avian Research Institute, Izatnagar, Uttar Pradesh, India

**Keywords:** cage free, egg, welfare, layer, chicken

## Abstract

India is ranked as the 2nd largest egg producer in the world. Despite the prevalence of backyard poultry (free range), a majority of the commercial egg-laying hens in the country are still housed in battery cages. There is a global shift toward cage-free eggs, due to regulations and increased demand from conscious consumers and food corporations. However, there are very few commercial cage-free facilities in India to meet this demand. The aim of this study was to undertake a needs-assessment survey of Indian egg producers on cage-free production, and understand what support is needed to build the capacities of the cage-free egg production sector to develop it into a viable and sustainable alternative to battery cage eggs. The results showed that nearly all producers agreed on the need for additional support in shifting to, and operating in, the cage-free sector. This included support in the form of financial assistance, technical training, and promotion of the cage-free sector. The results of this study highlight the pressing need for government and private support, in the absence of which cage-free producers are compelled to compete with battery cage poultry producers on prices, which will result in increased losses and failure of the sector, since they have not yet achieved economies of scale.

## 1 Introduction

Asia has been a global leader in egg production for decades, contributing to approximately 60% of the global production. In 2018, annual egg production in the region was 822 billion, through 3.1 billion layer hens (1). India has also seen tremendous growth, producing 138.38 billion eggs in 2022–2023, with the poultry market valued at INR 1905.3 billion, making India the 2nd largest egg producer in the world (1, 2). The sector is projected to continue its growth in the coming years, and is expected to reach INR 3477.8 billion by 2028 (3).

Much like the rest of the continent, layer hens (*Gallus domesticus*) used in the egg industry are housed in battery cages, i.e., barren wire-mesh cages housing 4–5 birds per cage. Battery cages have been widely challenged as cruel systems that provide inadequate housing for hens due to a lack of necessary space for movement, species-inappropriate flooring, and lack of opportunities to express natural behaviors, leading to physical and psychological suffering (4, 5). Recognition of the cruelty inherent to battery cages has also resulted in government bans on these barren cages, as seen in the European Union and several US states (6–8). Cage-free housing is recognized for providing better welfare due to increased space availability per bird and enrichment material and opportunities that facilitate the expression of natural behavior (9).

An increasing number of studies have documented a shift in consumer preferences toward higher welfare food products such as cage-free eggs, which ensure better living conditions for the animals involved (10–12). A growing concern for ethical consumption has also resulted in a shift in institutional consumption patterns, with thousands of food corporations, including those located in India, committing to use only cage-free eggs within the decade (13).

While corporations have made progress toward this goal in countries in the Global North, the rest of the world, particularly Asia, has seen very little progress toward cage-free procurement (14, 15). As of 2023, the Asia-Pacific (APAC) region has been found to have an average transition of 57% (16, 17).

This is, in large part, due to the infancy of the cage-free sector in Asia, and more specifically India. Most layer hens in India are still housed in battery cages, despite the prevalence of backyard poultry (free-range), particularly in periurban and rural areas (18). However, these poultry operate on a very small scale, with the eggs being consumed by the producers themselves or supplied to a few local families. There are very few cage-free farms that operate on a commercial level, as an alternative to the conventional battery cage facilities that are a prevalent practice in the country. The primary causes of the poor popularity of cage-free egg production in India are: lack of public and farmer awareness; an unorganized Indian market for cage-free eggs; a lack of technical information or HRD support; a lack of accountability for separating cage-free from non-cage eggs; Absence of government initiative, particularly in export assistance and market regulation. Given the increasing demand for cage-free eggs from conscious individuals and institutional consumers who are moving toward ethical sourcing, there is a pressing need for the growth of the Indian cage-free sector (3).

This study was undertaken to understand the perspectives of egg producers on the issue of cage-free systems, the key challenges in shifting to and operating in this sector, and the solutions and support required to overcome these barriers. The main goal was to understand the support needed to build the capacities of the cage-free egg sector in order to develop it into a viable and sustainable alternative for egg production in India.

## 2 Materials and method

### 2.1 Participants

Respondents were interviewed in October 2023 across the states of Karnataka, Telangana, Tamil Nadu, Andhra Pradesh, Uttar Pradesh, and Madhya Pradesh. The interviews were also recorded with the consent of the participants. The states covered in this study were selected on the basis of convenience sampling. Participants were approached by local collaborators who were familiar with the project topic, were given training in data collection, and briefed prior to conducting interviews. Since the cage-free sector in India is in its infancy, there are a limited number of producers who operate commercial cage-free facilities, limiting the sample size for the survey. Twenty egg producers across the country were engaged in this study. Out of these, 10 operate cage systems and 10 cage-free systems for egg production. The capacity of the farm, i.e., the number of laying hens reared by the respondents of the study included 50% having under 10,000 birds capacity; followed by 30% ranging 10,000–20,000; 10% with 20,000–50,000; and 5% each of 50,000–100,000 and above 100,000. It was seen recorded that the majority (70%) of the cage-free farmers had a capacity of under 10,000 birds with maximum cap of 20,000, whereas the cage farming respondents capacity ranged from under 10,000 to even over 100,000 bird capacity.

Participants were eligible to participate in the study if they:

- Gave their consent in writing, which was included on the questionnaire,
- engaged in commercial egg production,
- worked in the industry for at least 1 year, and
- operated in a managerial or ownership position at the facility.

### 2.2 Research tool

A combination of qualitative and quantitative methods were adopted for data collection and analysis. Separate questionnaires were designed for cage and cage-free producers. Kobo toolbox was used to create the questionnaire in English and translators were employed to convert the questionnaire in local languages- Hindi and Kannada. The answers were also collected using Kobo toolbox at each egg producing unit, through a network of local collaborators fluent in the respective native language. Responses in Hindi and Kannada were translated to English while filling the forms. The questionnaire consisted of 14–23 questions, as certain questions had follow-ups that would only apply if a specific answer was provided, excluding the producer’s contact details, facility name, and geographical location.

In this survey, we defined *cage-free farming* as a method of raising hens in non-caged housing, providing them with the freedom to move, stretch their wings and ideally access nest boxes, perches, foraging areas, and dust bathing spots. *Cage farming,* on the other hand, refers to the method of confining hens in small wire cages, typically in large numbers, where they are unable to exhibit their natural behaviors.

The questions that are relevant for our purposes are as follows:

1. *Most egg farmers in our country and around the world use cages. What are the reasons for using cages compared to cage-free systems? (Open-ended)*
2. *Some egg farmers are changing to cage-free systems. What do you think are the reasons to use cage-free rather than cage systems? (Open-ended)*
3. *What do you think are the biggest challenges and problems that prevent cage farmers from using cage-free systems? (Open-ended)*
4. *If an egg farmer decided to use a cage-free system what would be some of the solutions to the challenges outlined in the question above? (Open-ended)*
5. *If an egg farmer decided to use a cage-free system, would they need more support in the establishment or maintenance of the farm than is currently available? (Yes/No)*
6. *What support would they need? (Open-ended)*
7. *Who should offer that support? (Open-ended)*
8. *What are the main operational challenges in running your cage-free farm? (Open-ended)*

### 2.3 Data analysis

The data collected was analyzed using thematic qualitative analysis and descriptive quantitative statistics. All available responses were included in the analysis. Numerical data was analyzed using Microsoft Office tools.

## 3 Results

This study looks at the responses of egg producers to better understand what producers operating cage facilities perceive as the biggest challenges in shifting to cage-free systems, the real challenges experienced by those engaged in cage-free production, and the solution and support required to overcome the identified barriers. In seeking an answer to what support is required to build the capacities of the cage-free egg production sector to develop it into a viable and sustainable alternative to battery caged eggs, the results have been categorized into five themes:

1. Advantages of battery cage facilities;
2. Reasons to adopt cage-free systems;
3. Challenges in cage-free systems;
4. Potential solutions to challenges identified in cage-free systems; and
5. Support needed to transition to cage-free systems.

### 3.1 Advantages of battery cage facilities

*“Most egg farmers in our country and around the world use cages. What are the reasons for using cages compared to cage-free systems? (Open-ended)”*.

The most cited reason for preference for battery cage facilities is the ease in management of these facilities, in terms of providing vaccines and medication, maintaining biosecurity and controlling diseases, feeding, and egg collection. Lower costs of production was an additional factor for choosing caged systems. All responses are displayed in Table 1.

**Table 1:** Reasons stated for producers choosing caged systems over cage-free systems.

Theme identified	Factors	*n*	Percentage of responses
Economic considerations (33.33%)	Higher egg production	1	3.3
	Higher demand	3	10.0
	Industrial push toward cages	1	3.3
	Reduced egg breakage	2	6.7
	Automated feeding and watering	3	10.0
Health/Disease (33.33%)	Cleaner eggs	3	10.0
	Ease of medicines and vaccinations	3	10.0
	Reduced disease transmission	4	13.3
Investment required in caged production (26.67%)	Less space requirement	4	13.3
	Lower cost of production	1	3.3
	Less labor intensive	3	10.0
Hygiene concerns (6.67%)	Cleanliness	2	6.7

### 3.2 Reasons to adopt cage-free systems

*“Some egg farmers are changing to cage-free systems. What do you think are the reasons to use cage-free rather than cage systems? (Open-ended)”*

#### 3.2.1 Battery cage producers

Respondents operating battery cage facilities highlighted cost as a major factor in considering cage-free over cage systems. High infrastructure costs can make the establishment of battery cage facilities prohibitively expensive, taking into account the cost of the cages themselves, which one respondent shared was around Rs. 8–9 lakhs to house 5,000–6,000 birds. They also shared that when battery cage suppliers are located in other parts of the country, transportation costs add to the large investment required to set up these facilities. Wear and tear of the cages is also a cost addition, necessitating a replacement every 10–15 years. In comparison, cage-free facilities are a lot cheaper to establish.

#### 3.2.2 Cage-free producers

**Figure 1:**
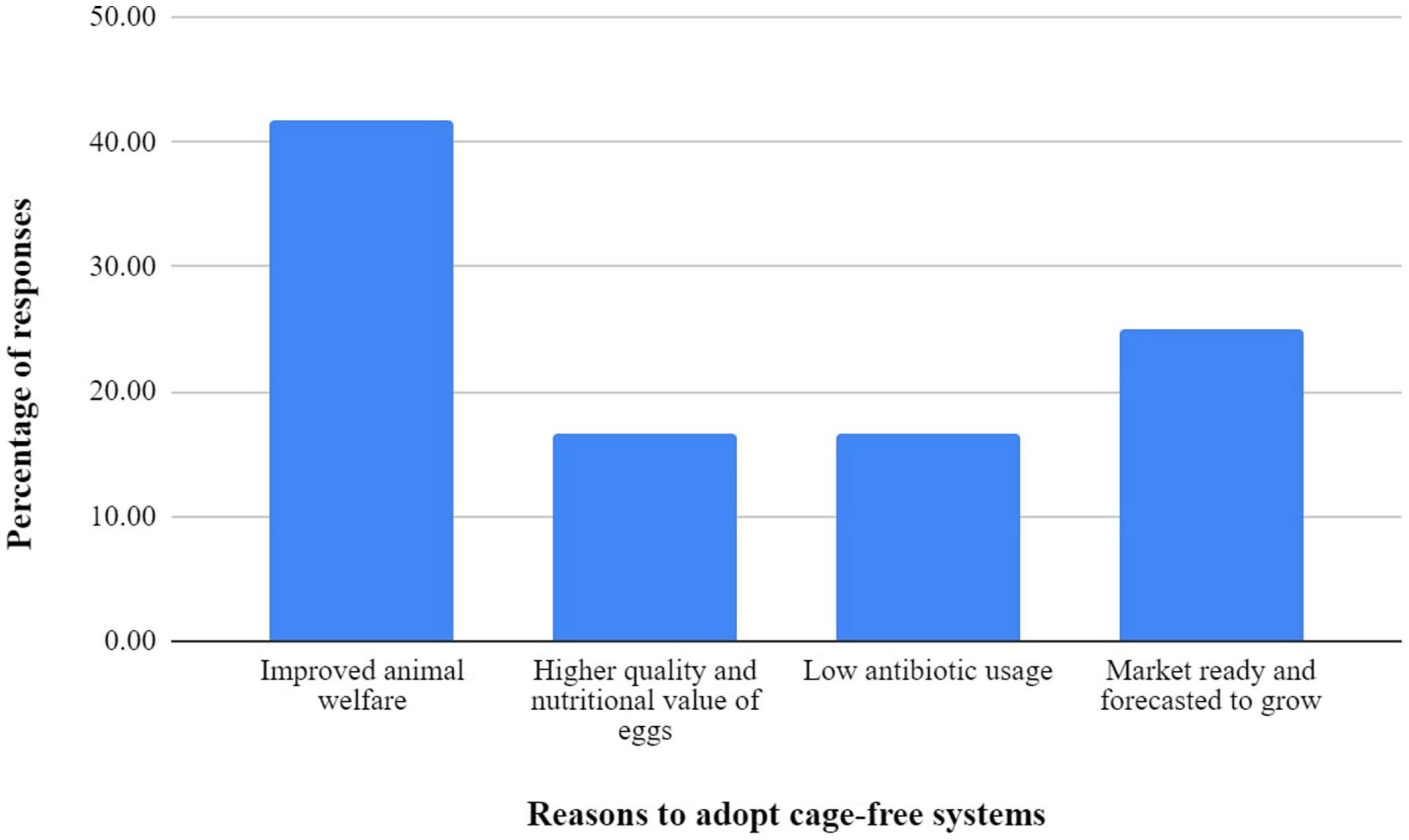
Responses of cage and cage-free producers regarding the rise of cage-free facilities.

The primary reasons that respondents cited for opting for cage-free facilities are the increased welfare of the layer hens, making this a more humane form of egg production, followed by the growing cage-free sector in the country. Respondents also preferred the ability to get higher and consistent prices year-round, as well as autonomy in deciding prices, as they are not dependent on the external parties. Some responses also highlighted the lower dependence on antibiotics and higher quality and nutrition of cage-free eggs. All the responses are displayed in Figure 1.

### 3.3 Challenges in cage-free systems

#### 3.3.1 Battery cage producers

*“What do you think are the biggest challenges and problems that prevent cage farmers from using cage-free systems?”*

When asked about the perceived challenges that may prevent the transition to cage-free systems, respondents operating cage systems were mainly concerned about the higher cost of production due to the presumption that cage-free facilities are more labor intensive and require a lot of land. They also cited greater challenges in feeding, watering, vaccination, medication and management of hens in cage-free facilities. Other concerns involved management, training and awareness, as well as the fear of lack of demand for cage-free eggs. Multiple respondents indicated the need to see a successful cage-free facility before considering a transition.

Many respondents also confused cage-free facilities with free range poultry facilities and believe hens to be vulnerable to animal attacks and diseases from migratory birds. All the responses are portrayed in Table 2.

**Table 2:** Ranking of the challenges perceived by battery cage producers in cage-free facilities.

Theme	***n***
High disease transmission	4
Frequent broken eggs	3
Difficult to monitor	3
Difficulty collecting eggs	3
Unclean eggs	3
Predator attacks	3
Space constraints	3
Difficult to vaccinate	2
Inadequate demand	1
High cost of production	1
Inadequate profits	2
Labor intensive	2
No precedent of large scale commercial cage-free farming	1

#### 3.3.2 Cage-free producers

*“What are the main operational challenges in running your cage-free farm”?*

The most pressing challenges identified were an increased resource dependence, including more labor and higher feed consumption. Lack of training in farm management for welfare and disease prevention was also a pressing concern. Additionally, a lack of awareness and market understanding about cage-free eggs is another challenge they faced. All the responses are portrayed in Table 3.

**Table 3:** Ranking of the main operational challenges faced by cage-free producers.

Theme	*n*
Large scale unviability	1
Low demand	1
Unclean and broken eggs	1
High space requirement	2
Lack of training	3
Labor intensive	3
High cost of production	3
Disease outbreaks	3

### 3.4 Potential solutions to challenges identified in cage-free systems

*“If an egg farmer decided to use a cage-free system, what would be some of the solutions to the challenges outlined in the question above? (Open-ended)”.*

The primary solutions identified to address the perceived challenges in operating cage-free systems included better training of staff, increased government assistance, improving consumer awareness, and better farm management practices. Respondents also highlighted the need to see successful large-scale cage-free facilities to fully understand how they operate and earn profits. All the responses are portrayed as an aggregate in Table 4.

**Table 4:** Suggested solutions to the challenges faced by egg producers in cage-free systems.

Theme	*n*
Employing more trained staff	1
Using nest boxes to prevent broken eggs	1
Litter management to prevent disease	1
Barricades (in free range)	1
Assistance from government	1
Farm monitoring	2
Preventative medication	2

### 3.5 Support needed to transition to cage-free systems

a *If an egg farmer decided to use a cage-free system, would they need more support in the establishment or maintenance of the farm than is currently available? (Yes/No)*

**Figure 2:**
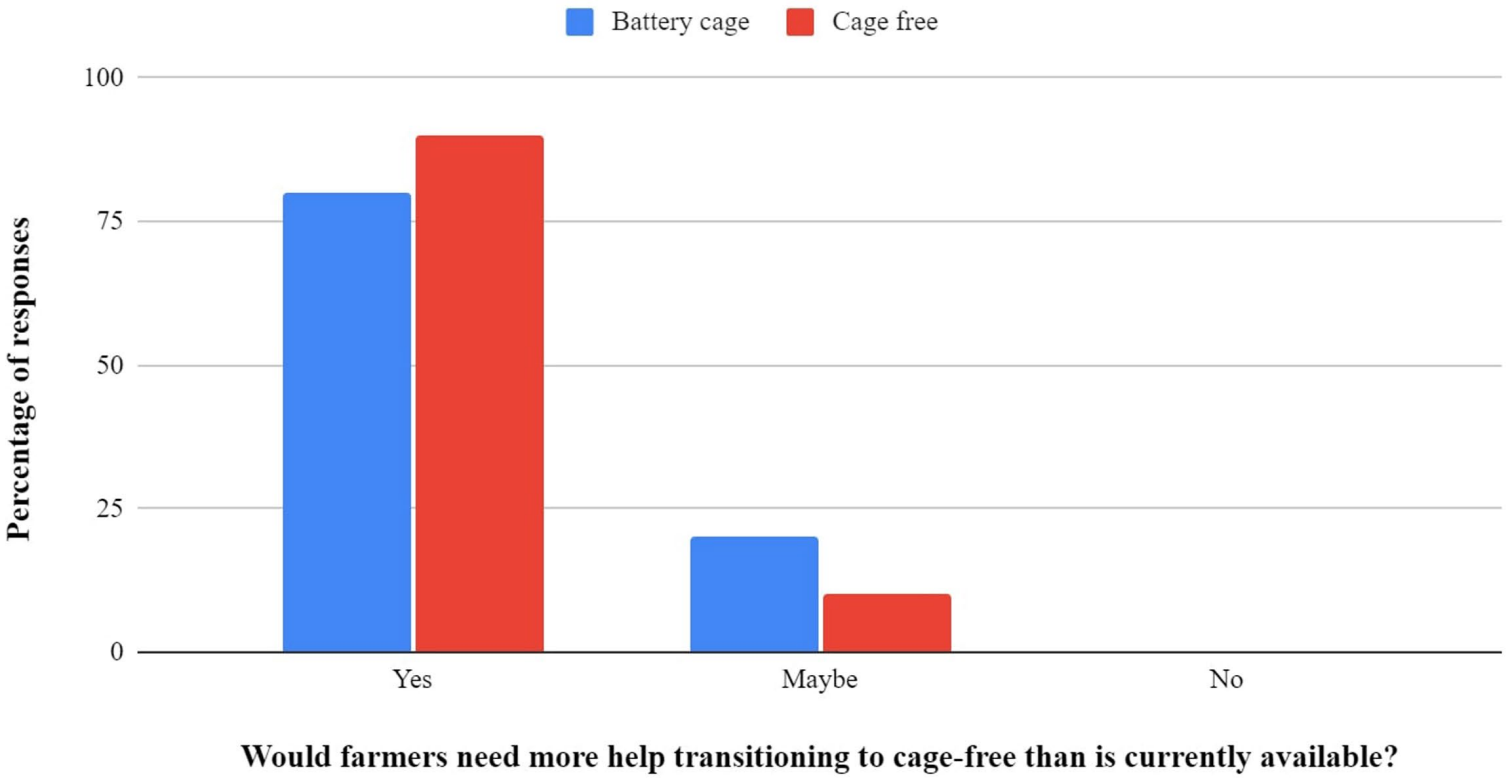
Cage and cage-free farmer’s responses to whether support is required for the establishment and maintenance of a cage-free farm.

The overwhelming response regarding the need for increased support for cage-free facilities was in the affirmative. This held true across both battery cage and cage-free respondents. Responses are displayed in Figure 2.

b *What support would they need? (Open-ended)*

**Figure 3:**
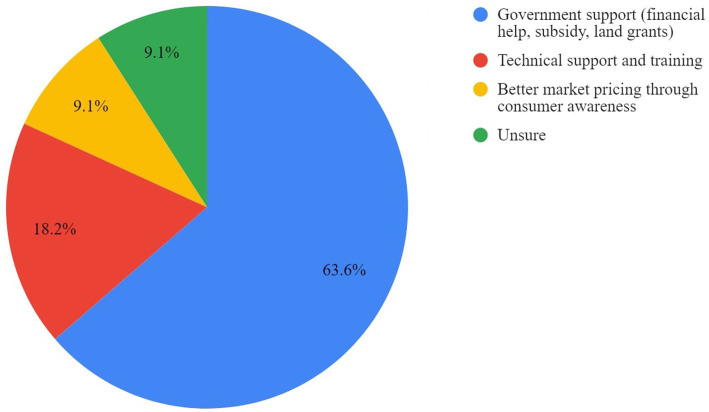
Support needed to transition to cage-free farming as perceived by cage producers.

**Figure 4:**
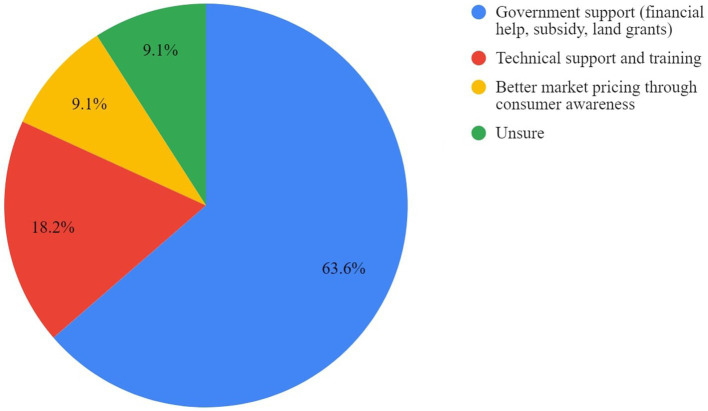
Support needed to transition to cage-free farming as perceived by cage-free producers.

Respondents identified financial assistance as the most important support to establish or operate cage-free facilities. Increased technical support in the form of training in management practices, market support through increased awareness, and uniform standards through certification were also highlighted. Responses are displayed in Figures 3, 4.

c *Who should offer that support? (Open-ended)*

**Figure 5:**
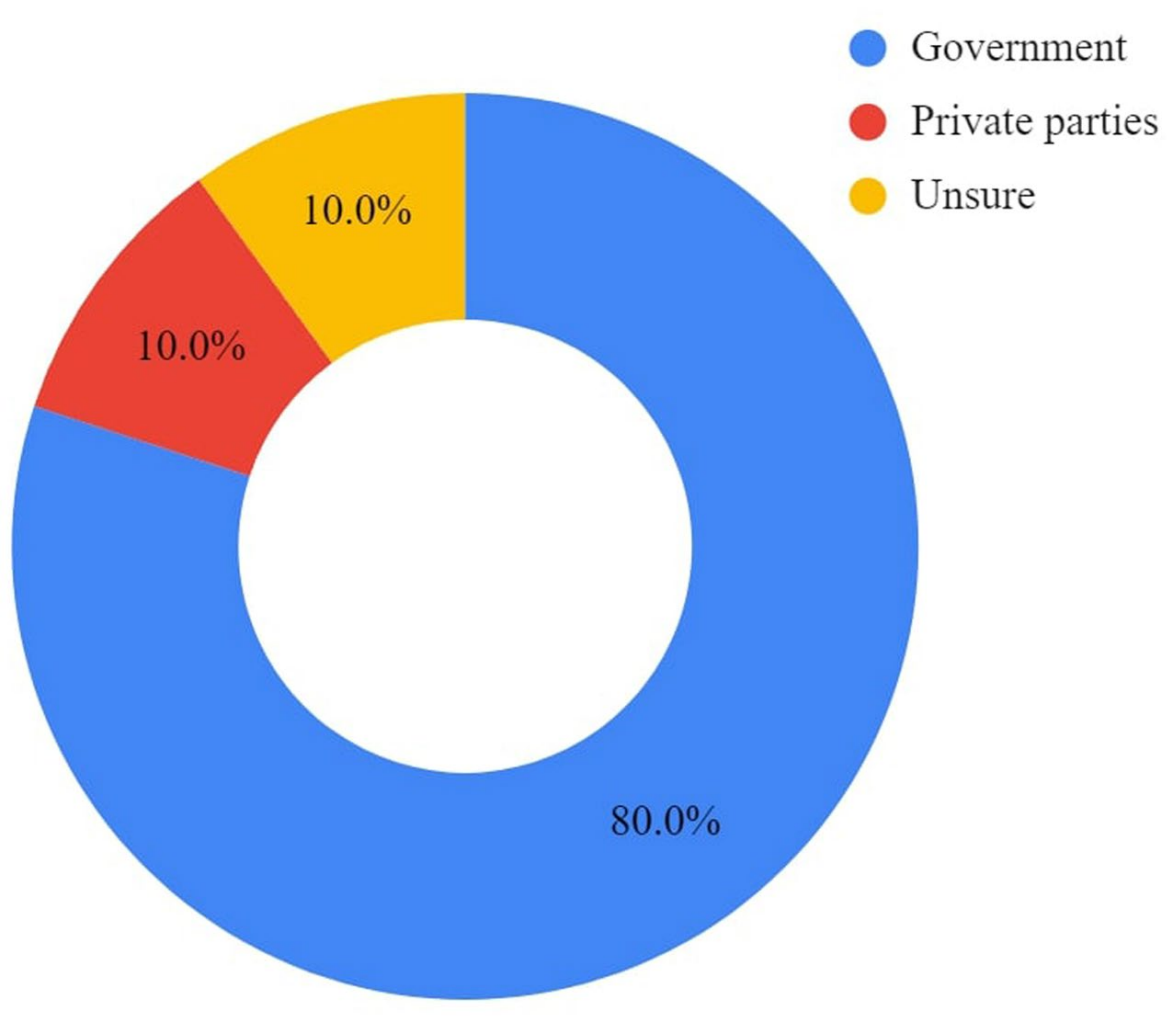
Cage farmers’ perspectives on who should offer support in transitioning to cage-free housing systems.

**Figure 6:**
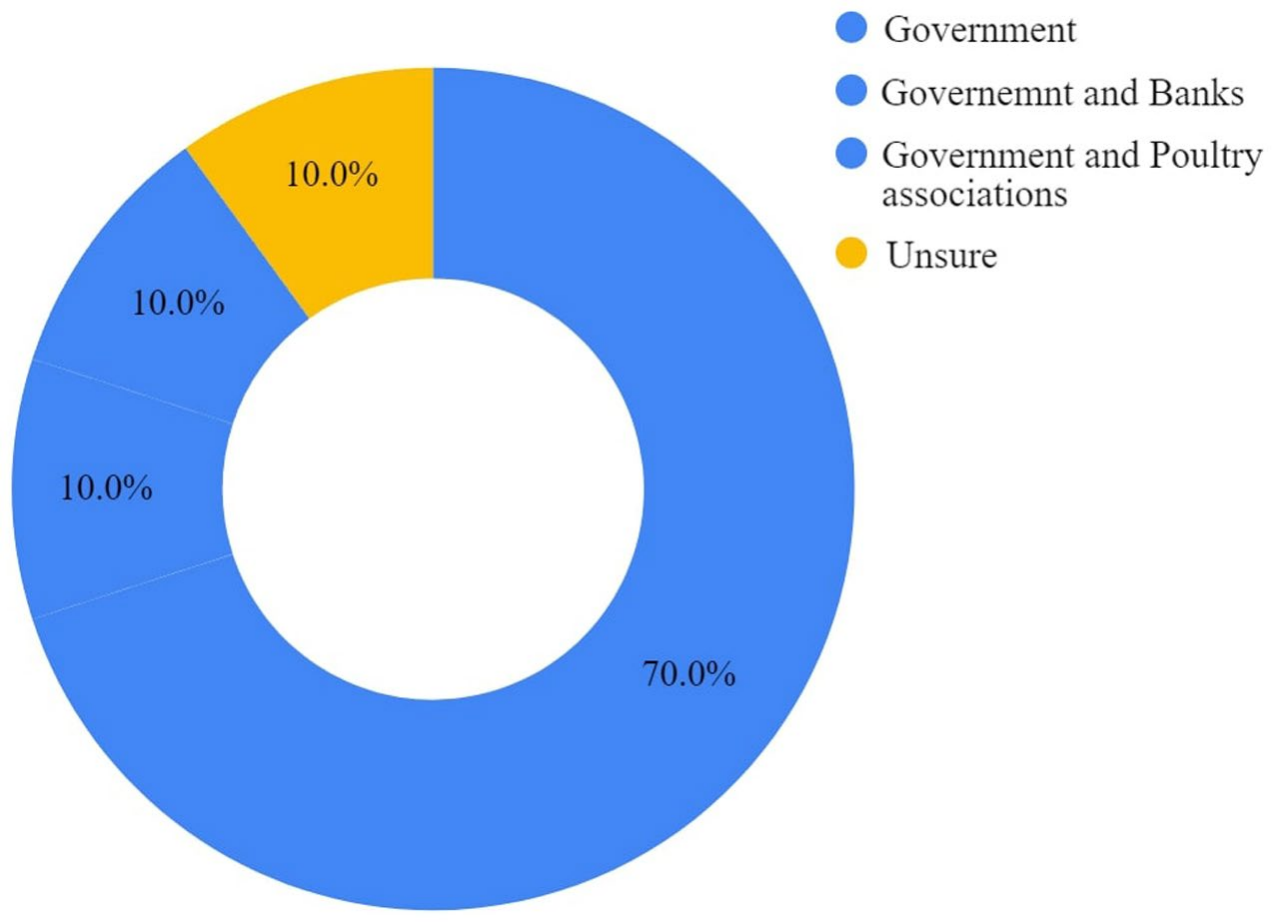
Cage-free farmers’ perspectives on who should offer support in transitioning to cage-free housing systems.

Both cage and cage-free respondents primarily identified the government as the body to offer increased support to establish and manage cage-free facilities. Some respondents also shared the need for support from other parties, such as banks, established poultry players, and poultry associations. All responses are displayed in Figures 5, 6.

## 4 Discussion

### 4.1 Reasons to adopt cage-free systems

The findings of this study present efficiency as the primary reason egg producers opt for cage systems, i.e., it is easier to provide feed, vaccinations and medication, and prevent diseases, with lower labor and land requirements. In the artificial conditions of cages, eggs are cleaner, less prone to breakage, and easier to collect.

However, high establishment costs are a major challenge to setting up caged facilities. The large expenses required in procuring cages, as well as the need to replace them every 10–15 years, was identified as a reason to opt for cage-free systems. A study found that although cage-free systems may potentially reduce profitability (19), it was made up with the possible cost savings from not requiring cage installations (20).

The present study found that caged egg producers in India are open to a possible transition to cage-free systems, and acknowledge certain advantages as well, but are largely constrained by a lack of available support. This is in line with past studies where producers have acknowledged the feasibility of cage-free transitions in other parts of Asia (20, 21). Respondents in this study also expressed not being confident about the commercial viability of cage-free systems due to an absence of large, profitable cage-free ventures to refer to as examples.

The primary reason stated for adopting cage-free systems was better animal welfare. It is widely recognized that there is an improvement in the welfare of laying hens in non-caged systems, in terms of both physical and psychological benefits (4, 22). Studies have also documented the reduction of pain suffered by hens in cage-free systems when compared to battery cage systems, finding that disabling pain is reduced by 63%, hurtful pain by 57%, and annoying pain by 70% (23).

Other driving factors were the increased consumer demand through corporate commitments, improved quality of eggs, and decreased use of antibiotics. Respondents operating cage-free systems highlighted better prices as an additional advantage, since they enjoy consistent prices throughout the year, and autonomy in deciding egg prices, with no dependence on external parties. All these benefits are a result of the continued growth of the cage-free sector in India, through increased consumer awareness, and demand at an individual and institutional level. A study conducted in 2022 highlighted a global increase in consumer awareness and concern for animal welfare in food production systems, with 71.9% of consumers from India agreeing that battery cage systems are cruel (12).

### 4.2 Challenges in adopting cage-free systems

In this study, the constraints perceived by cage producers in moving to cage-free systems include a higher cost of production, lack of awareness and training, increased land and labor requirement, and reduced profitability due to low demand. The other challenges listed were higher risks of disease outbreaks, difficulty in monitoring and record keeping, and higher incidence of unclean and broken eggs. A lack of awareness regarding the difference between free range/backyard poultries and cage-free systems also led to concerns about risk of attacks from predators, and spread of diseases from migratory birds.

In comparison, responses from cage-free producers also highlighted the issue of higher costs, attributed largely to increased feed consumption by birds that are allowed the freedom to move. However, they recognized that there is a growing demand for cage-free eggs by both individual and institutional consumers, countering perceived concerns about reduced profitability. This aligns with findings from other studies. Globally, there has been a rise in cage-free egg production following the 2012 EU ban directive (22), due to pressure from consumers regarding the welfare of layer hens. Consumers around the world support higher welfare eggs in the face of the cruelty experienced in caged facilities. A survey in Asian countries (20), stated 70–80% of egg consumers preferred that hens not suffer, and about 65–70% preferred cage-free eggs (12). The rising demand for cage-free eggs is evidenced through commitments made by food corporations to switch to higher welfare eggs within the decade (9).

Cage-free respondents aligned with caged producers on concerns about increased labor requirements, attributing it to a lack of automated systems in India’s nascent cage-free sector. Disease prevention was another common concern raised by respondents from both systems.

Aside from the above challenges, cage-free respondents were found to be deeply concerned about the lack of support structures for the sector, such as training opportunities, financial support, governmental recognition, and certification standards, which hinder their ability to operate and expand their scale of operations.

Other challenges envisioned by the caged producers regarding record-keeping, cleanliness, damaged eggs and unwanted behavior can be addressed through better management practices. Such problems were not faced by cage-free respondents in the current study, nor producers in other studies (20). Finally, one of the highlighted barriers raised by caged producers in the current study was a lack of land or space availability for shifting to cage-free production, which coincides with findings from other studies as well (20).

### 4.3 Solutions to the challenges

The main challenges in cage-free systems, as shared by respondents in the present study, were reduced profitability, higher production costs, disease outbreaks, and inadequate knowledge. These conclusions are similar to other studies conducted around the world (20, 24–26).

When asked for possible solutions to these challenges, respondents listed training opportunities and material, government support for finances, certification and labeling, and development of markets through awareness and education initiatives. These findings were in corroboration with other studies in Asia (10, 20, 27), in which egg producers in cage systems suggested that an increase in the sale of higher welfare eggs, along with supplementary measures like training in cage-free management and proper regulations, would lead to a smooth transition into producing cage-free eggs for a sustainable future (27). The need for increased public awareness to shift consumer preferences toward higher welfare eggs like cage-free has also been recognized, as there is low awareness about the conditions of laying hens in the egg industry (24).

Concerns raised by cage producers over unclean/broken eggs, difficulties in handling birds and preventing feather pecking, which were not corroborated by those engaged in the cage-free sector, can be addressed by better management (20). Improved farm management, facilitated through training programs and material, can address these issues. For instance, concerns about unclean and broken eggs are addressed by cage-free producers by utilizing nest boxes. Concerns about feather pecking are addressed by adding enrichment, such as pecking material, to the facility.

Cage-free housing is not in itself a cause for higher incidence of diseases. On the contrary, a report by the European Food Safety Authority found that caged production systems have a higher prevalence of salmonella, compared to non-caged systems (28). Effective management practices, such as monitoring the birds’ health, and timely administration of vaccines and medication can address concerns about disease prevention.

An additional challenge is the lack of adequate land availability which is a concern not just for participants of this study, but others as well. A transition to cage-free housing requires more space for each bird, which is a large factor in moving away from caged systems. However, establishing multi-level aviaries can address this issue - by increasing the number of hens housed in a given area, while maintaining adequate space requirements and welfare provisions (22, 31).

In the present study, some respondents could not offer any solutions to the barriers identified, and shared that the absence of success stories about large-scale commercially viable cage-free facilities makes it hard to envision such operations. There was a similar finding in another study (20, 27) where the respondents gave similar inconsistent responses in relation to commercial cage-free egg production. This highlights the need for model cage-free systems on a commercial scale, where producers can receive training on better management practices to improve productivity, welfare, and profitability. Additionally, these model facilities can also establish effective biosecurity measures to reduce disease prevention, and share the latest technologies and strategies to operate and manage cage-free facilities in a way that meets welfare and profit requirements.

### 4.4 Support needed in cage-free systems

In the present study, nearly all respondents across both categories believed that some form of support was necessary for establishing and operating cage-free systems. This can be broadly divided into- financial support, technical know-how, market support, and improved consumer awareness.

When asked who should provide such support, a majority of the respondents identified the government to provide support in terms of financial assistance, training and technical support, recognition and promotion of the cage-free sector, and introduction of standards for certification and labeling. Other sources of support were identified, such as banks (for financial support through low-interest loans, for instance) and poultry associations (for technical support such as management handbooks).

These findings align with past studies. One found that respondents pointed to support required from the government in terms of finance, training and extending awareness among consumers (29, 30). In another study, stakeholders reported increased consumer awareness and producer training as solutions for increased welfare-based egg production (30).

### 4.5 Applications

The findings of this study help in understanding the rationale behind the producers’ decisions toward adopting specific housing systems, the barriers in moving to (and operating) higher welfare systems, and the solutions required to do so. Through their input, this study has identified the need for financial support as well as its forms and sources; the gaps in technical knowledge on cage-free production methods and how they need to be bridged; and the market support required to ensure growth in this sector.

Input from stakeholders directly engaged in egg production is essential for informed decision-making regarding the promotion of higher welfare egg production. Accordingly, this study can serve as a resource to

- better understand the cage-free sector and its current limitations;
- understand the needs of egg producers when considering a shift to higher welfare forms of production;
- understand the support required to manage and grow existing cage-free operations;
- make policy decisions to support the cage-free sector in the country; and
- develop systems and materials to share technical knowledge;

### 4.6 Suggested initiatives

In light of the challenges, solutions, and forms of support shared by participants, as well as an analysis of other papers and studies, the following initiatives are likely to help the Indian cage-free sector grow:

- Development of management guide – Housing (for successful farming- Nine birds per sq. meter), nutrition, management details must be provided.
- Increased governmental financial support for cage-free production, in the form of subsidies, schemes, incentives, ease of business, and low interest loans.
- Certification and labeling standards for cage-free systems.
- Model facilities to provide on-site training for producers, and showcase the commercial feasibility of large-scale cage-free egg production.
- Increased technical support through training programs, manuals, and guides, and sharing technological advancements to improve management practices.
- Increased research to develop models, technologies, and methods to improve the efficiency of cage-free practices to make them more commercially feasible.
- Awareness programs to educate consumers and producers about cage-free systems, a sustainable and higher welfare model of egg production.

According on earlier research cites (20, 32), the following initiatives are recommended in the study to assist the Indian chicken industry in transitioning to sustainable, cage-free production:

Increase knowledge of the realities of effectively managed cage-free systems within the egg industry. On a big commercial basis. Encourage cooperation between local governments and egg producers in order to find appropriate land parcels for the pilot program of cage-free growing. Boost awareness and education about cage-free systems by creating training programs on best practices for managing them and inviting important stakeholders to participate. Pay particular attention to food safety, biosecurity, and efficient disease mitigation techniques.

## 5 Limitations

A negligible number of studies have been conducted in India analyzing cage and cage-free egg production. This study presents a starting point to conduct further in-depth research into this sector.

A significant limitation of this study is the small sample size, owing to the limited number of commercial cage-free facilities in the country, as this sector is in a nascent stage. Maintaining an even split between cage and cage-free respondents limited the sample size accordingly. However, the study provides insights into both real and perceived challenges regarding cage-free production, and the support required to overcome them. Another limitation is the large variability in the size of the poultry facility (2,200 to 1.35 lakh birds).

This study and the limitations therein highlight the need for an additional comprehensive exploration of the cage-free sector in India, and the needs of the producers and consumers.

## 6 Summary of economics

⟡ Cage-free egg production is an intensive system of rearing as the farmers in Indian is following stocking density range from 11 to 12 birds/m^2^ for pullets and 9 to 10 birds/m^2^ for adult birds.
⟡ At international level ideal stocking density should be 6.17 birds/m^2^ (European legislation), 7.15 birds per m^2^ (Global Animal Partnership, 2017), 6.2 birds per m2 (AGW Animal Welfare Approved), The German label “Für mehr Tierschutz” (standard and premium) allows a maximum stocking density of 7 birds/m^2^.
⟡ It is different farming from the basic backyard farming where we gave access of foraging to the birds but in Cage Free rearing birds are stall fed for lifetime without any opportunity of foraging. It’s nothing but commercial intensive layer farming in deep litter with enrichment facilities, and we should not confuse with Backyard small scale farming.
⟡ The Cage free farmers participated in our survey have minimum of 2,220 birds and maximum 20,000 bird’s capacity farm. We strongly feel for good commercial output minimum of 1,000 birds stock is needed
⟡ Based upon our study we have come up with following model of 1,000 birds-

1. Birds capacity- 1000
2. Average efficiency of production – up to 92%
3. Average egg production of birds- 240-260 eggs per year
4. Mortality- 7-10%
5. Cost of Production of one egg- 6.5 Indian rupees per egg (including recurring and non-recurring cost)
6. The cost of packaging, storage and transport per egg- 0.5 to 0.7 Indian Paisa
7. The cost of Sale- 12-20 Indian rupees per egg depending upon branding.
8. Pure Profit per egg - Varying from 3 to 7 Indian rupees per egg

## 7 Conclusion

As consumers in the Indian sub-continent increasingly prioritize ethical considerations in their purchasing decisions, the egg production industry needs to adapt to meet these evolving expectations. The transition to cage-free production represents a significant stride toward creating a more ethical and sustainable future for egg production. While acknowledging the positive aspects of cage-free production, it is essential to recognize the challenges associated with this transition. A significant insight that emerged from this study was that in the absence of adequate aid from the government, cage-free producers are compelled to compete with battery cage poultry producers on prices, which will result in increased losses and failure of the sector.

The current study is aimed at understanding the reasons and challenges in considering the adoption of cage-free systems. The possible solutions and types of support were also discussed, and applications were suggested on the basis of the results. The exploration of cage-free production in India underscores the interconnectedness of animal welfare, industry sustainability, and consumer preferences. While the challenges are significant, they are likely to be addressed as technology, research, and industry expertise continue to advance. This will pave the way for more widespread adoption of cage-free systems, as seen in other mature markets such as Europe and the United States.

As we move forward, it is imperative for stakeholders in the egg production sector, including producers, policymakers, and consumers, to collaborate in fostering an environment where ethical and sustainable practices are not only encouraged but are also economically viable. The evidence presented in this document suggests that cage-free production holds promise not only in meeting the growing demand for ethically produced eggs but also in shaping a more compassionate and resilient food production system.

## Data availability statement

The raw data supporting the conclusions of this article will be made available by the authors, without undue reservation.

## Author contributions

JR: Writing – review & editing, Writing – original draft, Investigation, Conceptualization. AC: Writing – original draft, Investigation, Data curation. NS: Writing – review & editing, Validation, Supervision, Project administration. PW: Writing – review & editing, Validation, Project administration, Methodology, Data curation. MM: Validation, Writing – review & editing, Methodology. DB: Data curation, Writing – review & editing. AT: Writing – review & editing, Visualization, Resources, Funding acquisition.
